# Effective Delivery of Endogenous Antioxidants Ameliorates Diabetic Nephropathy

**DOI:** 10.1371/journal.pone.0130815

**Published:** 2015-06-26

**Authors:** Yongsoo Park, Hyunok Kim, Leejin Park, Dongsoo Min, Jinseu Park, Sooyoung Choi, Moon Hyang Park

**Affiliations:** 1 Department of Internal Medicine and Bioengineering, Hanyang University College of Medicine and Engineering, Seoul, Korea; 2 Department of Biomedical Science and Research Institute of Bioscience and Biotechnology, Hallym University, Chunchon, Korea; 3 Department of Pathology, Hanyang University College of Medicine, Seoul, Korea; University of Pittsburgh, UNITED STATES

## Abstract

**Background:**

Diabetic nephropathy (DN) is thought to be partially due to the injury of renal cells and the renal micro-environment by free radicals. Free radial scavenging agents that inhibit free radical damage may well prevent the development of underlying conditions such as mesangial expansion (by inhibiting extracellular matrix expression) in these patients.

**Methods:**

Using techniques for intra-cellular delivery of peptides, we made metallothionein (MT) and superoxide dismutase (SOD), potent endogenous antioxidants, readily transducible into cell membrane and tested their protective effect against the development of DN in OLETF rats. Herein, we study antioxidant peptides for their ability to prevent oxidative damage to primary rat mesangial cells (MCs), which are important constituents of renal glomeruli.

**Results:**

Intraperitoneal administration of these antioxidants resulted in delivery to the kidney and decreased ROS and the expression of downstream signals in renal cells and postponed the usual progression to DN. In *in vitro* experiments, MT and SOD were efficiently transferred to MCs, and the increased removal of ROS by MT and SOD was proportional to the degree of scavenging enzymes delivered. MT and SOD decreased three major oxidative injuries (hyperglycemia, AGE and ROS exposure) and also injuries directly mediated by angiotensin II in MCs while changing downstream signal transduction.

**Conclusions:**

The protective effects of MT and SOD for the progression of DN in experimental animals may be associated with the scavenging of ROS by MT and SOD and correlated changes in signal transduction downstream. Concomitant administration of these antioxidant peptides may prove to be a new approach for the prevention and therapy of DN.

## Introduction

Microvascular injuries associated with diabetic nephropathy (DN) is the primary etiology for end-stage renal disease [1, 2]. Oxidative stresses related to metabolic dysregulation in diabetes affect various tissues leading to many various diabetic complications [3–6]. Overproduction of reactive oxygen species (ROS) leads to oxidative injury of the cellular and extracellular components of the kidney, especially in mesangial cells (MCs) [3, 4]. It results in an increase in substrates for advanced glycation end products (AGEs) and in precursors of glycoxidation and lipoxidation products, and accelerates the free-radical formation that may be accompanied by, or caused by, a deficiency of antioxidant and detoxification pathways [5–8]. The manner by which elevated oxidative stress alters downstream signaling leading to the evolution of clinical DN is still not elucidated and it is still not known if antioxidant supplementation can help the damaged renal tissues to regenerate and alleviate this life-threatening complication of diabetes [5, 8, 9].

DN is characterized by mesangial expansion (ME) and glomerular basement membrane (GBM) thickening [4, 10]. ME results from coordinated alterations of not only extracellular matrix (ECM) regulatory enzymes (matrix metalloproteinases (MMPs)) and tissue inhibitors of these MMPs, but also ECM constituent peptides such as types I and IV collagen, fibronectin, and laminin, together with proliferation signals like TGF-β1, CTGF and angiotensin II (AGII) [4, 9, 11]. MCs are important constituents of mesangial spaces and appear to act as important mediators of ME, mostly as a result of oxidative injury [10]. AGII is also found to be an important cause of the progressive injury in DN [7, 12]. Intrarenal AGII exerts most of its well-known effects by binding to type 1 AGII (AT1) receptors that are abundant in the cells of the glomeruli, including MCs, tubules, vasculature, and interstitium. AT1 receptor activation causes an increase in vascular resistance and a resulting decrease of renal blood flow, leading to the production of ECM in the mesangium and tubulointerstitium [7, 9].

As described in detail previously, metallothionein (MT) is a highly inducible protein that binds heavy metals and plays an important role as a potent antioxidant due to its many cysteine residues [13–15]. Superoxide dismutase (SOD), another major class of antioxidant enzyme involved in controlling levels of ROS, catalyzes the destruction of the O_2_^-^ free radical [14, 15]. MT and SOD, endogenous antioxidants have the potential to protect cells and tissues against oxidative stress such as diabetes and diabetic complications but they have limited ability to cross lipid bilayers, so it is not clear that they have similar antioxidative effects *in vivo*. Recently, applying the HIV-1 Tat protein called cell penetrating peptide (CPP) [16–18], we have succeeded to deliver these antioxidants into living cells including pancreas and nerves and to prevent both the development of diabetes itself and diabetic neuropathy [13–15]. Using this same CPP technology, the in-frame Tat-MT and Tat-SOD fusions were demonstrated to efficiently transduce into MCs through the phospholipid membrane to prevent hyperglycemia-, AGE-, ROS- and AGII-induced cellular injuries. We also found that intraperitoneally injected Tat-MT and Tat-SOD in combination could transfer to renal tissue *in vivo* alleviating DN in Otsuka Long-Evans Tokushima Fatty (OLETF) rats.

## Materials and Methods

### Ethics Statement

This study was carried out in strict accordance with the recommendations in the Guide for the Care and Use of Laboratory Animals of the National Institutes of Health (NIH, publication No. 85–23 m, revised 1996). The protocol was approved by the Institutional Animal Care and Use Committee at the University of Hanyang (Permission Number: HY-IACUC-09-021). All appropriate means were undertaken to minimize suffering.

### Cloning, expression and purification of fusion proteins

By way of inserting a fusion of the Tat nucleotide sequence with either the genes for SOD, MT or GFP into the prokaryotic pRSET expression vector (Invitrogen, Carlsbad, CA), we produced a fusion peptide of the basic domain (amino acids 49–57) of the HIV-1 Tat with each construct as previously described [13–15]. The Tat-MT, Tat-SOD and Tat-GFP fusion products were translated in *E*. *coli* BL21 (DE3) pLysS (Novagen, Madison, WI) and isolated by Immobilized Metal Affinity Chromatography (IMAC) using a column of Ni-NTA resin (Bio-Rad, Hercules, CA). Remaining salts were removed from the purified 6-His-tagged protein via PD10 column chromatography (Amersham, Buckinghamshire, UK). Each of these expression products contained a sequence encoding six histidine residues and TAT (YGRKKRRQRRR). MT without Tat and SOD without Tat were also made and purified similarly, as described previously [15]. All constructs were verified by sequencing.

### Experimental Protocol

Tokushima Research Institute (Otsuka Pharmaceutical, Tokushima, Japan) graciously provided us with male OLETF and Long-Evans Tokushima Otsuka (LETO) rats at 4 weeks which were kept at controlled temperature (23 ± 2°C) and humidity (55 ± 5%) with a 12 h light/dark cycle. The rats were allowed free access to standard rat chow. Fluid requirements were provided with 30% sucrose solution thereby accelerating onset of diabetes and diabetic complications. LETO controls (n = 10) received only drinking water without 30% sucrose solution. Around the age of 20–24 weeks, the diabetic state of the OLETF rats was confirmed by obtaining successive random blood glucose levels that exceeded 13.9 mmol/l. We first checked the renal penetration of a single intraperitoneal administration for each of the fusion proteins in animals at 20 weeks of age (4 days after transduction), after the diagnosis of diabetes mellitus was confirmed. Then, since antioxidants in combination were superior to either antioxidant alone in increasing cell viability *in vitro*, all the diabetic OLETF rats were randomly segregated into three groups: OLETF rats without transduction group (n = 10), a Tat-GFP group (n = 9) and a Tat-MT-Tat-SOD combination group (n = 8). The Tat-GFP rats were injected with 3 mg/kg of Tat-GFP every 3 days, and the antioxidant combination group with 3 mg/kg of Tat-MT and 3 mg/kg of Tat-SOD every 3 days. The fusion protein treatment continued for 16 weeks. At the conclusion, all animals were sacrificed using ketamine, with subsequent rapid removal of kidney tissues for morphology and biochemical examination.

### Measurement of urinary protein

Metabolic cages were used to collect urine from each experimental animal over a 3 day period. Pooled urine samples centrifuged for 10 minutes at 3,000g and supernatants were evaluated using the Bradford method for protein and an ELISA kit was used to quantify microalbumin (Bethyl Laboratories, Montgomery, TX). Measurements were performed on each rat just before (0 week) and 16 weeks after beginning transduction. Assays were performed in triplicate expressing mean values of protein and microalbumin as the daily amount of urine excreted by a given rat.

### Histological examination

Tat-fusion protein therapy was given for 16 weeks, at which time the kidneys were carefully dissected from the euthanized animals to prevent any damage. The removed organs were then rinsed with phosphate-buffered saline (PBS, pH 7.2), weighed and fixed in 10% buffered formaldehyde solution (pH 7.4). The kidneys were then dehydrated using serially graded alcohol solutions, and set in paraffin. 4μm slices obtained from a Leica RM 2145 microtome were stained using Periodic acid-Schiff (PAS) and Masson`s trichrome stains for histopathological analyses. The sections were then photographed under an Olympus photomicroscope (Tokyo, Japan) at the X400 magnification and measurements of glomerular or mesangial area were made. Glomerular area was measured in 20 completely round glomeruli of four animals per group, randomly chosen from all renal cortices. Each area positively stained for the respective staining was calculated after manually tracing each glomeruli with the automated Image-Pro Plus software (Media Cybernetics, Bethesda, MD). Ultra-thin (70 nm) sections of the kidney were also qualitatively investigated using a Zeiss 10 (Zeiss, Oberkochen, Germany) transmission electron microscope. For ultra-thin sectioning, the kidney tissues were fixed in 4% glutaraldehyde and 0.1% sodium phosphate (pH 7.4) for 4 h at 4°C. Then, the specimens were washed twice in phosphate buffer and post-fixed in 1% osmium tetroxide in the same buffer at 4°C overnight. Samples were dehydrated in an ethanol series and embedded in Spurr resin. Sectioning was carried out with a Potter Blum MT1 ultramicrotome. The GBM thickness and the width of the foot processes along with the degree of ME were measured from the electron micrographs at ×20,000 magnification studied at 20 different randomly selected sites in four animals per group. Measurements of GBMs were made at each point where a line on the grid intercepted an endothelial-GBM interface and the thickness was measured on a line orthogonal to the edge of the GBM at the endothelial side of the intercept. The widths of the foot processes on the peripheral GBM or filtration surfaces were also measured at 20 different randomly selected sites in four animals per group and the average foot-process width was calculated. The degree of ME was also measured with the automatic image analyzer, using the same Image-Pro Plus software.

### Immunohistochemical analysis

4 μm paraffin sections mounted on silanized slides, were dewaxed using xylene before rehydration in graded alcohol. Microwave antigen retrieval was performed in citrate buffer (pH 6.0) for 10 min. Sections were incubated with 1% H_2_O_2_ for 30 min to block residual endogenous peroxidase and then water-washed followed by blocking with normal goat serum for 30 min at room temperature. After slides had been incubated overnight at 4°C with nitrotyrosine specific primary antibodies (Millipore, Temecula, CA), α-SMA, collagen IV (Ventana Medical Systems Inc., Tucson, AZ), TGF-β (V), VEGF (A-20) and CTGF (H-55) (Santa Cruz Biotechnology, Santa Cruz, CA) at 1:200 dilutions immnuoreactivity was analzyed by incubating with horseradish peroxidase conjugated goat-anti-rabbit IgG antibody for 30 min at room temperature. 3,3`-diaminobenzidine (DAB) was used to detect peroxidase activity using DAB kits obtained from Vector Laboratories (Burlingame, CA). Slides were mounted after hematoxylin counterstainng, and then rinsed with tap water. After being dehydrated using xylene, sections were mounted and then photographed under a photomicroscope at a magnification of X400. At least 20 glomeruli per section were observed and analyzed for the percentage distribution.

### Western blotting

Kidney tissue and whole cell extracts were incubated in lysis buffer (150 mmol/l NaCl, 50 mmol/l Tris-Cl, 1 mmol/l EDTA, 1 mmol/l PMSF, 1 mmol/l Na_3_VO_4_, 1 mmol/l NaF, 1% NP-40, 0.25% deoxycholic acid and 1 μg/ml leupeptin) for 20 min on ice, followed by 15 min centrifugation at 1500 rpm. Extracts in loading buffer were electrophoresed on 8~12% acrylamide gels and the proteins were transferred onto PVDF membranes in transfer buffer for 1 h at 120V. Membranes were blocked for 1 h in TBST [20 mmol/l Tris-HCl (pH 7.6); 137 mmol/l NaCl; and 0.1% Tween 20] with 5% skim milk, and then incubated overnight at 4°C with one of the following antibodies: His-probe (H-15), MT (N-19), SOD-1, NF-κBp65 (F-6), total LRP6, total GSK-3β (H-76), phospho-GSK-3β (Ser 9), β-catenin (H-102), VEGF (A-20), CTGF (H-55), α-tubulin and β-actin (Santa Cruz Biotechnology, Santa Cruz, CA); phospho-p38, phospho-ERK, phospho-JNK, phospho-AMPKα (Thr172), phospho-LRP6 (Cell Signaling Technology, Beverly, MA); fibronectin (Sigma, St. Louis, MO); RAGE from Abcam (Cambridge, MA). Membranes were thoroughly washed with TBST and then incubated with horseradish peroxidase conjugated secondary antibody at 1:20000 dilution (HRP-linked goat anti-rabbit IgG; Santa Cruz Biotechnology) washed in TBST and detected with an ECL system (iNtRON Biotechnology, Ansan, Korea).

### Reverse Transcriptase-polymerase chain reaction (RT-PCR)

RNA from primary cultured MCs or whole kidneys was isolated using TRI Reagent (iNtRON Biotechnology, Ansan, Korea). Two μgm of RNA/20 μl reaction volume were combined with random hexamer primers (2.5 μmol/l) and reaction components for 30 minutes at 42°C and then heated to 95°C for 4 minutes to inactivate the enzyme and to denature RNA-cDNA hybrids. cDNA amplification was performed at a final concentration of 1X DNA polymerase reaction buffer, 1.5 mmol/l MgCl_2_, 200 μmol/l deoxynucleoside triphosphates (dNTP), 10 pmol/l of target primers or β-actin primers, and 1.25 U of AmpliTaq DNA polymerase (Promega Co., Madison, WI) in a total volume of 40 μl. The primer sequences are shown in S1 Table. The amplification procedure involved denaturation at 95°C for 1 min, primer annealing at 55°C for 45 sec and extension at 72°C for 30 sec. Final PCR products were electrophoresed in 1% agarose gels containing 0.2 g/mL ethidium bromide. β-actin gene expression served as control for the RT-PCR. RT-PCR results were quantified by densitometric analysis using Quantity One 1-D Analysis Software (Bio-Rad, Hercules, CA). Target mRNA expression was calculated by normalizing with respect to β-actin mRNA expression [19].

### Electrophoretic mobility-shift assays (EMSA)

As described in detail previously [13], NF-κB activation was assayed by gel mobility-shift assays of nuclear extracts. A NF-κB consensus oligonucleotide (Promega, Madison, WI) was used in the electrophoretic mobility shift assays. Bound and free DNA was resolved by electrophoresis on a 6% native polyacrylamide gel in 89 mmol/l Tris–HCl, 89 mmol/l boric acid, and 2 mmol/l EDTA.

### Zymography (Measurement of MMP-9)

To analyze gelatinolytic activity, aliquots of culture medium were mixed with 5 μl 5X non-reducing sample buffer (0.5 mol/l Tris-HCl (pH 6.8), 50% glycerol, 0.5% bromphenol blue). Electrophoresis was performed on 8% acrylamide gels containing 1 mg/ml gelatin as substrate. Then, the gels were incubated in 2.5% Triton X-100 and 50 mmol/l Tris-HCl (pH 7.5), for 1 h at room temperature, followed by overnight at 37°C in a collagenase buffer containing 50 mmol/l Tris-HCl (pH 7.5), 100 mmol/l NaCl, and 10 mmol/l CaCl_2_. The gels were then stained with Coomassie blue, and zones of lysis were visualized. Proteolysis was identified as a white zone in a blue background and the Kodak Gel Logic 100 imaging system used to document the results (Eastman Kodak, Rochester, NY).

### Cell culture and experimental conditions

Primary cultured MCs were used in this study. Kidneys from ether-anesthetized SD rats (weighing 170 g) were obtained using sterile procedure. Kidney cortices were isolated after removing the capsules an then minced with a razor blade to a fine paste, and then pressed through a set of stainless steel sieves (Nos. 140, 80, and 200; Fisher Scientific Co., Pittsburgh, PA). Glomerular rich portion was collected from the top of the 75 μm sieve, which resulted in >98% pure glomeruli. The glomeruli were pelleted for resuspension in DMEM (Invitrogen, Carlsbad, CA) supplemented with 20% FBS and antibiotics (100μg/ml streptomycin, 100U/ml penicillin). The glomerular suspension was plated in tissue culture flasks and incubated at 37°C in 5% CO_2_. Primary cultures of MCs were allowed to grow for 3–4 week, reaching confluency during that time. The MCs were used between the 7th and 10th passage. For individual experiments, cells were seeded into six-well plates at a density of 2 x 10^5^ cells per well in complete DMEM medium for 24 h. To transduce the Tat fusion proteins into cultured MCs, 1 μmol/l of the appropriate protein was added to the culture medium for 1 h. Following transduction, the medium was replaced by serum-free DMEM containing one of the various experimental oxidative stress inducing agents.

To study high glucose-, AGE- and 4-hydroxynonenal (4-HNE) (Calbiochem, La Jolla, CA)-induced injury by ROS, cells were plated in six-well plates in serum-free DMEM and exposed to one or other damaging agent. To examine the ability of Tat-MT and/ or Tat-SOD to protect against high glucose-induced injury, hyperglycemia was induced by exposing the MCs to high glucose (30 mmol/l) DMEM for 24 h together with 1 μmol/l Tat fusion protein. Cells exposed to low glucose (5.5 mmol/l) served as control. To examine the capacity of Tat-MT and/ or Tat-SOD to protect against AGE-induced injury, MCs were exposed to 400 μg/ml of AGE in low glucose with 1 μmol/l of Tat fusion protein. Control cells were exposed to 400 μg/ml of BSA. The production of AGE has been described previously [13]. To examine the ability of Tat-MT and/ or Tat-SOD to protect against 4-HNE-mediated injury, the cells were exposed to 40 μmol/l of 4-HNE with 1μmol/l of Tat fusion protein for 24 h.

### Confocal microscopy

We used indirect immunofluorescence assays and confocal microscopy to localize proteins. Briefly, MCs grown on cover slips in 12-well plates were treated with the fusion proteins for 1 h. After washing in PBS, the cells were fixed with 4% cold paraformaldehyde for 30 min and permeabilized with 0.2% Triton X-100. Rabbit poly-histidine, MT and SOD antibodies (Santa Cruz Biotechnology, Santa Cruz, CA) were applied for 1 h followed by incubation with fluorescein isothiocyanate-conjugated goat anti-rabbit IgG for 1 h. Post-incubation, the cells were placed in the chamber of an Olympus microscope for observation with a confocal laser-scanning system. Fluorescence images (excitation 494 nm/emission 518 nm) of the cells were recorded every 0.25s (×400).

### Statistical analysis

Data are presented as means ± SEM. Differences in mean values were tested by Student’s t-test using SPSS for Windows (version 18.0; SPSS, Chicago, IL). Differences in clinical parameters including urinary protein measurements were analyzed by one-way or repeated measures of analysis of variance (ANOVA). P values < 0.05 were considered statistically significant.

## Results

### Transduction of antioxidants into MCs

Similar to our previous experiments [13, 14], our fusion proteins, Tat-MT, Tat-SOD and Tat-GFP (A-C in S1 Fig) were purified by chromatography following translation in *E*. *coli*.To study whether Tat-MT, Tat-SOD or Tat-GFP fusion proteins could be transduced into MCs, we added each protein to the culture medium for 1 h, and examined the intracellular expression of poly-histidine by Western blotting and confocal microscopy. As shown in D-E in S1 Fig, Tat-GFP, Tat-MT and Tat-SOD were efficiently transduced into the MCs. After 1 h incubation with 1 μmol/l of Tat-fused proteins, confocal microscope examination showed that nearly all of the cells were positively stained for poly-histidine. Incubation with MT or SOD alone did not result in cellular uptake. The Tat-fusion proteins persisted as long as 4 days after transduction (data not shown). Intracellular presence of the exogenously administered Tat-MT and Tat-SOD inside MC cells was verified by staining with MT and SOD antibody, respectively. Fluorescence was detected in both cytoplasm and nucleus.

### *In vivo* transduction of antioxidant peptides affects the development of DN

**Fig 1:**
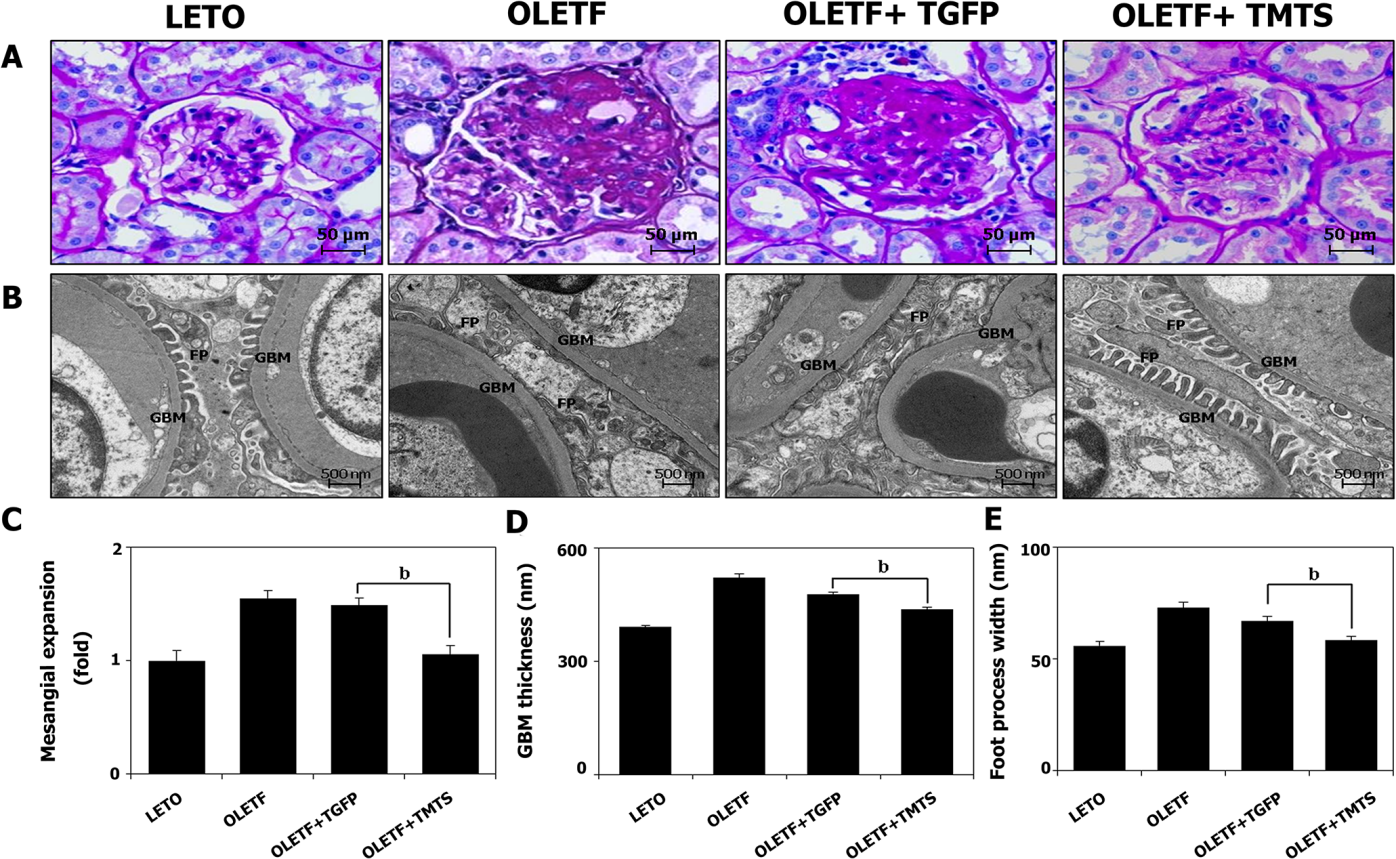
Changes in renal histology after antioxidant treatment for 16 weeks. Diabetic OLETF rats at 20week of age were injected *i*.*p*. either with treatment of 3mg/kg of Tat-GFP or the same amount of antioxidants in combination biweekly. (A) Representative renal histologic findings demonstrated by Periodic acid Shiff (PAS) staining (magnification X400); (B) Electron micrograph of glomerulus (magnification X20000); (C) Quantitative analysis of images of PAS-stained kidney sections; (D) Quantitative analysis of images of GBM thickness via electron micrographs. (E) Quantitative analysis of foot process width via electron micrographs. Values are means±SEM (n = 8 rats in each group). ^b^ *p*<0.01 vs. Tat-GFP treated group. Abbreviations; TGFP: Tat-GFP, TMTS: Tat-MT-Tat-SOD combination.

We examined whether DN, which developed in nearly all the OLETF rats, could be delayed or inhibited by administration of Tat-fusion proteins. First, the ability of the Tat fusion proteins to enter renal tissues was tested. 3 mg/kg of Tat-fusion proteins were given via a single intraperitoneal injection to OLETF rats at the age of 20 weeks. First, we demonstrated successful TaT fusion protein transduction into renal tissues dissected from OLETF rats 4 days post-intraperitoneal injection of Tat-GFP or antioxidants in combination (F in S1 Fig). Then we investigated the antioxidant protein effects on development of DN, by treating a different group of rats with 3 mg/kg of either Tat-GFP or antioxidants in combination every 3 days for 16 weeks starting at 20 weeks of age. LETO rats or OLETF rats without transduction were used as histological controls. Body weight, fasting plasma glucose or insulin concentrations did not differ between the OLETF rats with and without antioxidant treatment. Importantly, 24 hour urine microalbumin excretion increased in the OLETF rats without transduction and Tat-GFP-treated OLETF rats, while it decreased significantly in the antioxidant-treated OLETF rats. Changes in the level of 24 hour urine protein excretion did not differ between different treatments in accordance with the changes in kidney weight (Table 1). Fig 1 illustrates typical histologic changes seen in the treated rats. Control OLETF rats displayed the usual ME pathology marked by ECM accretion and capillary wall thickening. Subsequent analyses quantified that the PAS-positive areas, important indices of ME, were significantly larger in the Tat-GFP-treated OLETF rats (and in the OLETF without transduction) than in the antioxidant-treated OLETF rats (and in the control LETO). Mesangial matrix expansion in the Tat-GFP-treated OLETF rats (and in the OLETF without transduction) was reduced in the group receiving antioxidants (Fig 1A and 1C). More detailed ultrastructural findings were obtained from ultra-thin sections viewed with a transmission electron microscope (Fig 1B). In the Tat-GFP treated OLETF rats (and in the OLETF without transduction), in addition to the thickening of GBM and ME, the foot processes of podocytes were fused and exhibited the typical diabetic ultrastructural features of broadening of foot processes and podocyte effacement. GBM thickness and foot process width were significantly decreased in the antioxidants treated group and nearly the same in comparison to the LETO rats of the control group (Fig 1D).

**Table 1:** Changes in the level of clinical and renal parameters induced by transduction of Tat-fusion proteins for 16 weeks.

	LETO		OLETF		OLETF+TGFP		OLETF+TMTS	
Clinical Characteristic	0 week	16 week	0 week	16 week	0 week	16 week	0 week	16 week
Body weight (g)	508.40±7.59	545.80±13.68** ^,^ ^‡^	617.20±8.59	770.65±19.62^†^	616.38±9.08	732.11±20.56*	606.81±11.79	729.00±30.66**
Fasting plasma glucose (mg/dl)	90.20±13.15	95.20±11.17** ^,^ ^‡^	122.70±13.05	135.30±6.29	120.77±12.68	136.44±10.00	120.62±17.79	132.75±10.43
Plasma insulin (ng/ml)	1.79±0.27	1.89±0.25	2.07±0.43	1.70±0.29	2.08±0.38	1.72±0.22	2.04±0.36	1.74±0.67
Kidney weight (g)	-	2.94±0.11^#^ ^,^ ^##^	-	5.00±0.69	-	4.87±0.52	-	4.33±0.69
Urine protein (mg/ml)	69.60±5.28	144.50±12.36** ^,^ ^‡^	172.42±20.23	279.79±34.74	161.80±17.83	264.47±25.82	160.90±12.37	234.32±22.13* ^,^ ^$^
Microalbumin (μg/day)	10.21±4.71	17.36±2.17** ^,^ ^‡^	18.69±3.85	40.82±4.36	17.36±3.83	40.06±7.07	16.82±4.12	31.51±3.18^#^ ^,^ ^†^ ^,^ ^$$^

Diabetic OLETF rats were randomly divided into two groups, a Tat-GFP treatment group and a Tat-MT-Tat-SOD combination treatment group. The former were injected every 3 days with 3 mg/kg of Tat-GFP, and the latter with 3 mg/kg of Tat-MT and 3 mg/kg of Tat-SOD. The fusion protein treatment continued for 16 weeks. LETO rats were used as histological controls. Urinary protein levels were determined by the Bradford method and microalbumin levels were measured with a microalbumin quantitation ELISA kit (Bethyl Laboratories, Montgomery, TX).

Values are expressed as means±SEM.

*p<0.05 vs. OLETF

**p<0.01 vs. OLETF

^†^p<0.05 vs. OLETF+Tat-GFP

^‡^ p<0.01 vs. OLETF+Tat-GFP by repeated measures of ANOVA.

^#^p<0.05 vs. OLETF

^##^p<0.01 vs. OLETF

^$^p<0.05 vs. OLETF+Tat-GFP

^$$^p<0.01 vs. OLETF+Tat-GFP by one-way ANOVA.

Abbreviations; TGFP: Tat-GFP, TMTS: Tat-MT-Tat-SOD combination.

### Expression level of inflammation, fibrosis and oxidative stress markers after antioxidant treatment

**Fig 2:**
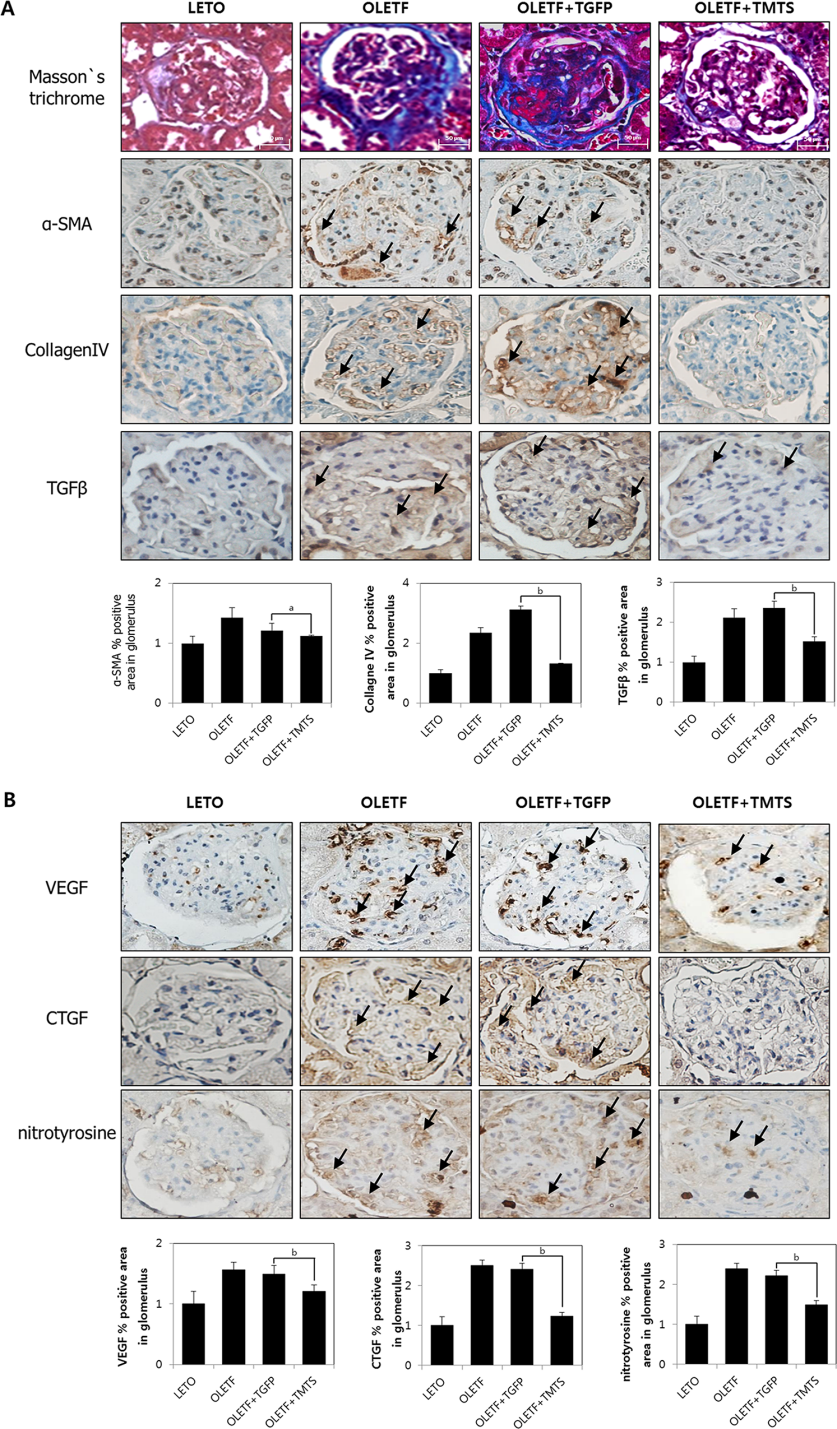
Amelioration of renal fibrosis, histological damage and level of ROS after antioxidant treatment. Diabetic OLETF rats were treated as in Fig 1. LETO rats or OLETF rats without transduction were used as histological controls. After 16 weeks of transduction, kidney tissues were removed for respective staining. (A) Representative figures of Masson’s trichrome, α-SMA, collagen IV, and TGF-β1staining (magnification,×400) with and without 16 weeks of antioxidant transduction; (B) Representative figures of VEGF, CTGF, and nitrotyrosine staining (magnification,×400) with and without 16 weeks of antioxidant transduction. Arrows point to positively stained areas. Glomerular area posivitve for respective antibodies were quantified with Image Pro Plus software.Values are means±SEM (n = 8 rats in each group). ^a^ *p*<0.05,^b^ *p*<0.01 vs. Tat-GFP treated group. Abbreviations; TGFP: Tat-GFP, TMTS: Tat-MT-Tat-SOD combination.

Quantitative analyses also confirmed that not only the glomerular Masson’s trichrome, α-SMA, and collagen IV stain-positive areas, but also TGF-β, VEGF, CTGF and nitrotyrosine stain-positivity, important indices of ME, inflammation, oxidative stress and fibrosis were larger in the Tat-GFP-treated OLETF rats (and in the OLETF without transduction) than in the antioxidant-treated OLETF rats (and in the control LETO). Treatment of the OLETF rats with antioxidants in combination significantly decreased Masson’s trichrome positivity and the area of glomeruli positive for nitrotyrosine and TGF-β stain (Fig 2).

**Fig 3:**
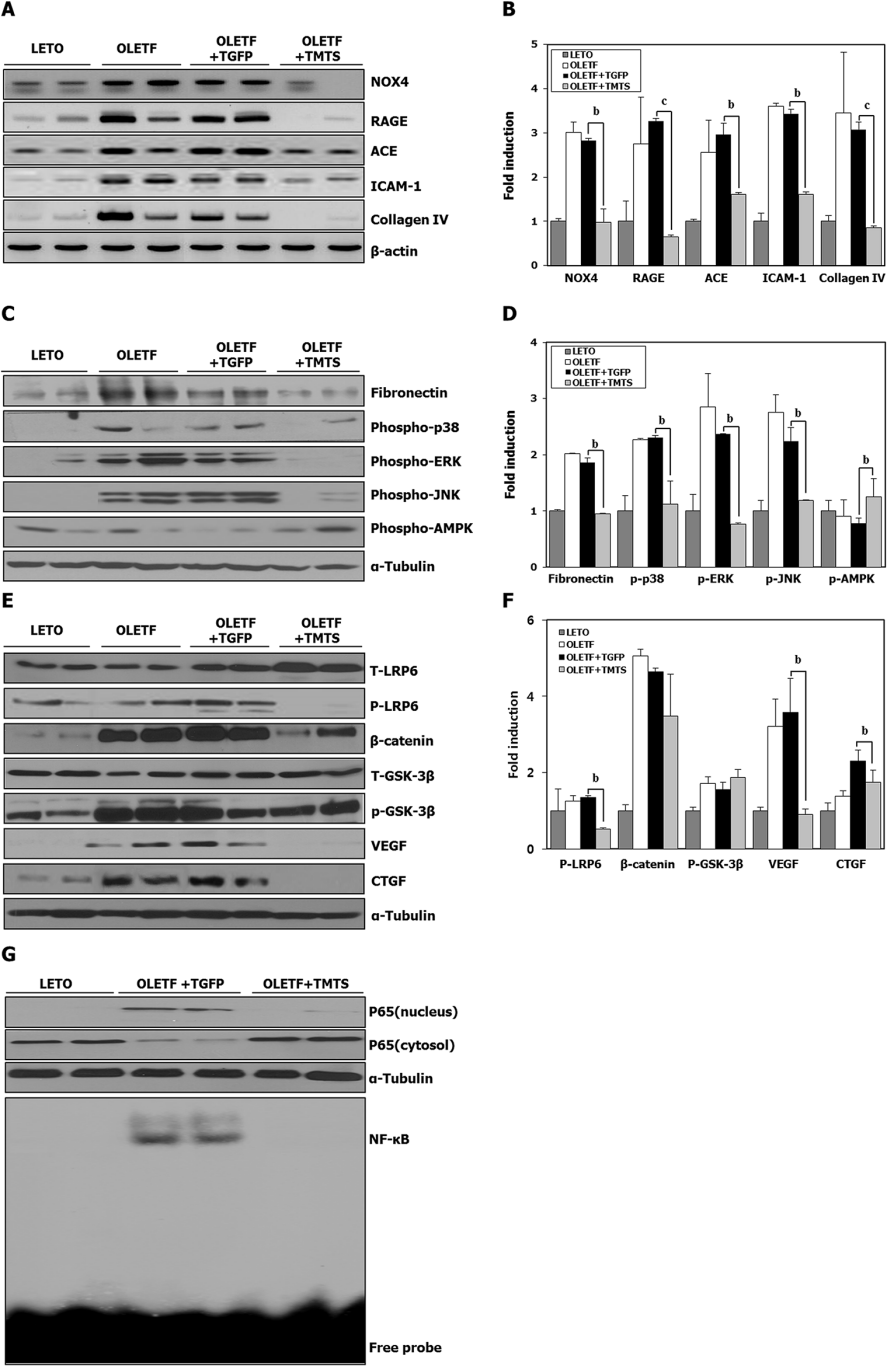
*In vivo* expression of oxidative stress, inflammatory and fibrosis signaling molecules after 16 weeks of transduction. Diabetic OLETF rats were treated as in Fig 1. LETO rats or OLETF rats without transduction were used as histological controls. After 16 weeks of transduction, kidney tissues were removed for RT-PCR (NOX4, RAGE, ACE, ICAM-1 and collagen IV) (A, B) and Western blotting analysis (fibronectin, phospho-p38, phospho-ERK, phospho-JNK, phospho-AMPK, total and phospho-LRP6, β-catenin, total and phospho-GSK3, VEGF, CTGF) (C-F). β-actin mRNA and α-tubulin protein served as loading controls (n = 8 rats in each group). (G) A representative immunoblot for NF-κB p65 using nuclear proteins from kidney tissues and an EMSA. Nuclear extracts were mixed with a double-stranded ^32^P-labeled oligonucleotide encoding the decameric consensus sequence of NF-κB and separated by PAGE. Values are means±SEM (n = 8 rats in each group). ^b^ *p*<0.01, ^c^ *p*<0.001vs. Tat-GFP treated group. Abbreviations; TGFP: Tat-GFP, TMTS: Tat-MT-Tat-SOD combination.

Renal tissues were isolated 16 weeks after initiation of the Tat-fusion protein treatment and processed for RT-PCR and Western blotting (Fig 3). Renal tissues of rats exposed to antioxidants revealed less evidences of inflammation and fibrosis (decreased RAGE, ACE, ICAM-1, collagen IV, fibronectin, VEGF and CTGF expression) along with less expression of NOX4, MAPK (p38, ERK and JNK) and NF-κB. Interestingly, renal expression of AMPK appeared to increase in antioxidant-treated rats. In addition, expression of β-catenin and WNT proteins that are upregulated in renal tissues of the Tat-GFP treated OLETF rats (and in the OLETF without transduction) tended to decrease in the antioxidant treated rats (Fig 3E). Taken together, our results show that structural alterations of the renal glomeruli and tubulointerstitium occur in conjunction with the physiological changes in multiple redundant ROS-mediated signaling mechanisms brought about by treatment with antioxidants.

### Antioxidant administration affects expression of inflammatory cytokines in primary cultured MCs

**Fig 4:**
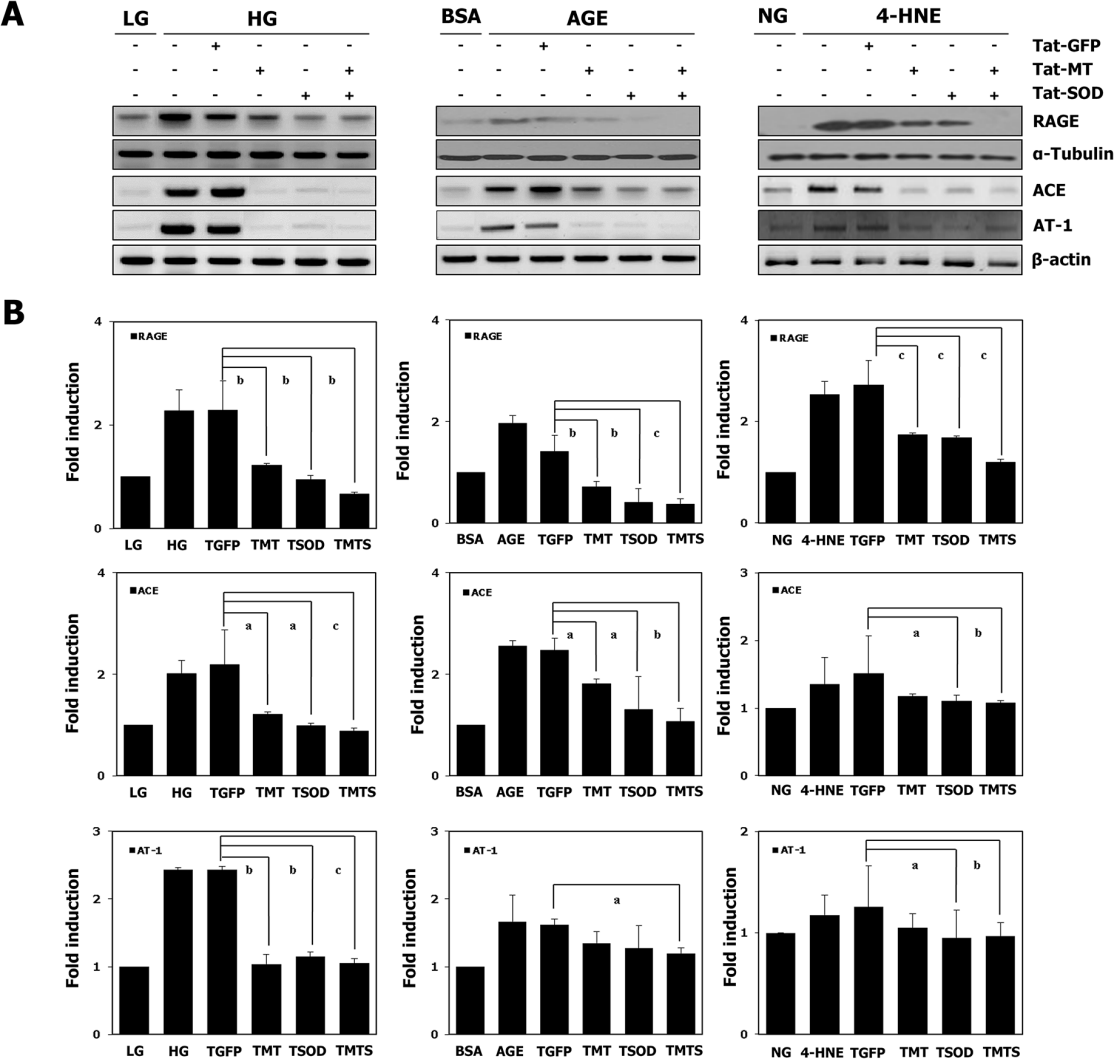
Influence of antioxidants on RAGE and angiotensin II expression in MCs after various injuries. Primary cultures of MCs were transduced with 1 μmol/l Tat-GFP, Tat-MT, Tat-SOD, or a combination of Tat-MT (1 μmol/l) and Tat-SOD (1 μmol/l), and subsequently transferred to DMEM with high glucose (30 mmol/l) for 24 h, exposed to AGE (400 μg/ml) for 24 h, or exposed to 4-HNE (40 μg/ml) for 24 h. Cells in the control group were incubated in low glucose (5.5 mmol/l) DMEM, 400 μg/ml of BSA or normal glucose (11.1mmol/l) DMEM respectively. (A) RAGE and angiotensin II expression in MCs was investigated by Western blotting (RAGE) and RT-PCR (ACE, AT-1). Protein-matched cell extracts were probed for RAGE using polyclonal antibody and α-tubulin served as loading control. Data are representative of three experiments performed on different days. (B) Quantification of the Western blotting and RT-PCR results. Western blots were quantified by densitometry and are expressed as fold changes relative to controls. RT-PCR results were quantified by densitometric analysis using Quantity One 1-D Analysis Software (Bio-Rad, USA). Target mRNA expression was calculated by normalizing with respect to β-actin mRNA expression. Data are expressed as mean ± SEM (n = 9). ^a^ *p*<0.05,^b^ *p*<0.01, ^c^ *p*<0.001vs.the Tat-GFP treated group. Abbreviations; LG: low glucose (5.5 mmol/l), NG: normal glucose (11.1 mmol/l), HG: high glucose (30 mmol/l), TGFP: Tat-GFP, TMT: Tat-MT, TSOD: Tat-SOD, TMTS: Tat-MT-Tat-SOD combination.

**Fig 5:**
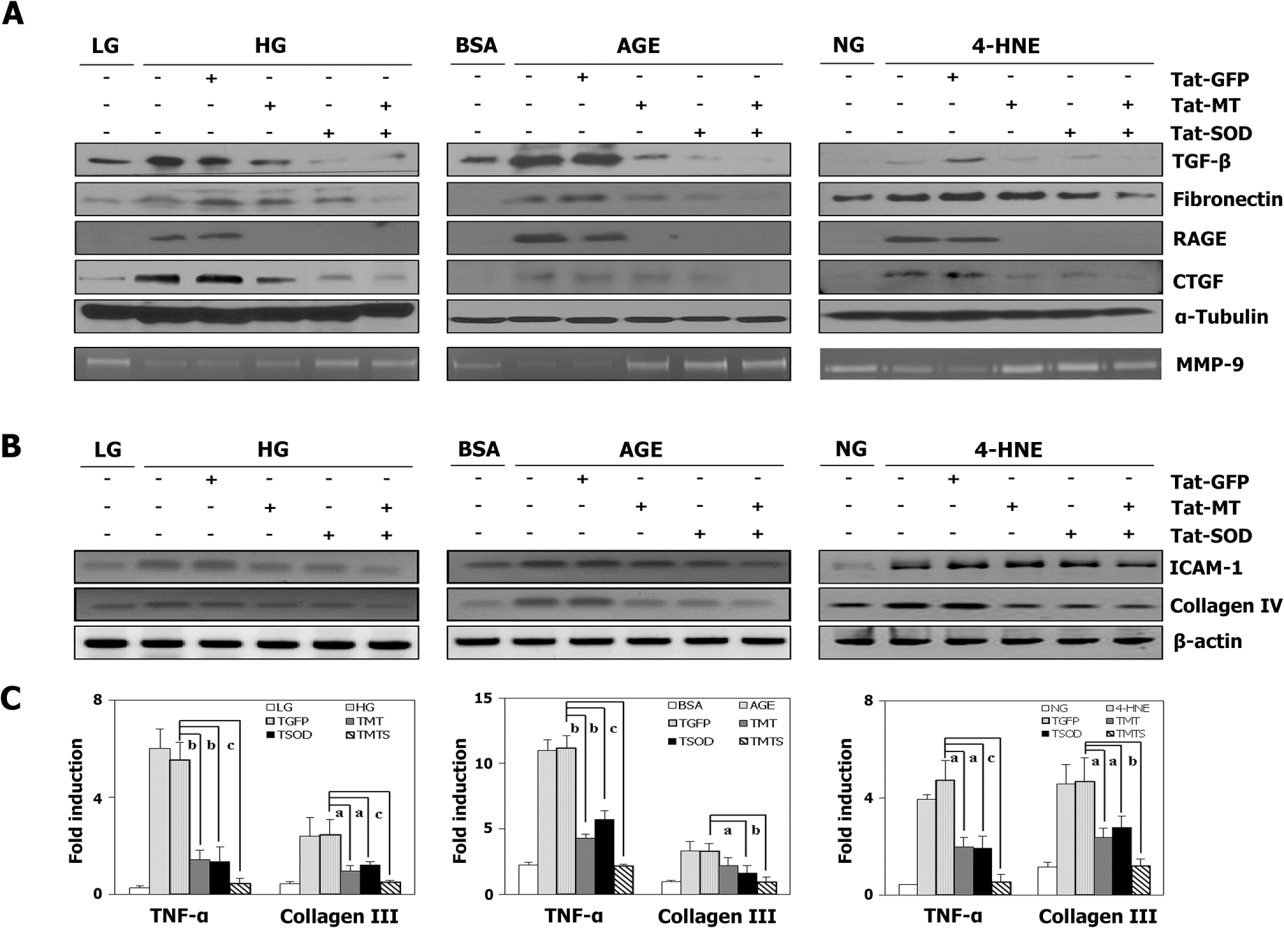
Effects of fusion proteins on inflammatory protein expression in MCs after various injuries. (A) Inflammatory molecular expression in MCs was investigated by Western blot analyses (TGF-β, fibronectin, RAGE, CTGF), and zymography (MMP-9). Protein-matched cell extracts were probed for fibronectin with polyclonal antibody and α-tubulin served as loading control. Data are representative of three experiments performed on different days. (B) Inflammatory molecule expression in MCs was investigated by RT-PCR (ICAM-1, collagen IV), and (C) real-time PCR (TNF-α, collagen III). Abbreviations; LG: low glucose (5.5 mmol/l), NG: normal glucose (11.1 mmol/l), HG: high glucose (30 mmol/l); TGFP: Tat-GFP, TMTS: Tat-MT-Tat-SOD combination. Data are expressed as mean ± SEM (n = 3). ^a^ *p*<0.05,^b^ *p*<0.01, ^c^ *p*<0.001vs.the Tat-GFP treated group.

We then demonstrated that transduction of the fusion protein constructs, Tat-MT, Tat-SOD or Tat-GFP, did not show any significant cytotoxicity (data not shown). We also tested their effects on levels of expression of inflammatory molecules after injury induced by high glucose, AGE or 4-HNE. As shown in Fig 4, the different types of injuries were found to increase expression of RAGE and AGII levels in MCs as indicated by the expression of ACE and AT-1. Treatment with Tat-MT, Tat-SOD, or Tat-MT and Tat-SOD in combination reduced RAGE and AGII dramatically. Intracellular activity of MT and SOD increased in a dose dependent manner (S2 Fig), and Tat-MT (1 μmol/l) and Tat-SOD (1 μmol/l) in combination were superior to either antioxidant alone in decreasing RAGE and AGII expression (Fig 4). The increases in RAGE activation, ACE and AT-1 mRNA expression after exposure to high glucose, AGE or 4-HNE were completely prevented by transduction of the two antioxidant proteins. The expression of RAGE increased by as much as 3 fold with high glucose exposure for 24 h, while antioxidant treatment decreased expression to 0.9 ~ 1.9 fold the control level. When MCs were exposed to AGE for 24 h, antioxidant supplementation decreased RAGE expression from 3.5 fold to 0.7 ~ 1.2 fold the control level. Damage induced by 4-HNE, increased the RAGE expression by as much as 2.5 fold the control level, and combined antioxidants treatment decreased it to 1.1 ~ 1.7 fold that of controls (Fig 4). As shown in Fig 5, all the different types of injuries increased expression of the downstream oxidative stress, inflammatory and fibrosis molecules (RAGE, TGF-β, fibronectin, collagen IV, ICAM-1 and CTGF) while antioxidant treatement decreased them in MCs. As expected, they decreased the activity of MMP-9 and treatment with Tat-MT and/ or Tat-SOD increased its expression. The increased expression of TNFα and collagen III after different injuries, measured by real-time RT PCR was also found to be blocked by the treatment of antioxidants. In general, the effects of antioxidants in combination were superior to those of either antioxidant alone. We also investigated whether the antioxidants could dampen the oxidative stress and inflammation induced by AGII itself. As expected, AGII dose-dependently increased ROS formation and inflammatory molecule expression in rat MCs, either due to hemodynamic perturbation or metabolic derangement, while Tat-MT significantly reduced these effects (S3 Fig). Collectively, these observations indicate that antioxidants delivered by protein transduction are effective in reducing ROS and protecting renal cells from inflammation induced by hyperglycemia, AGE, 4-HNE and AGII itself.

### Antioxidants decreased multiple redundant ROS-mediated signaling mechanisms in MCs

We then demonstrated antioxidant effects on the activity of MAPK such as p38, ERK and JNK. ROS produced by the various damaging conditions increased phosphorylation of p38, ERK and JNK, while Tat-MT, Tat-SOD and their combination nearly abolished this phosphorylation. We also studied the effect of antioxidants on the activity of AMP kinase, an important energy sensor in various cellular environments. The various ROS-inducing injuries reduced expression of AMP kinase and the antioxidant combination appeared to oppose this effect. Interestingly, all the ROS producing injuries activated WNT signaling, while antioxidants inhibited it (S4 Fig). The only exception was pGSK3β, which did not change by the treatment of antioxidants.

NF-κB is also involved in causing insult to cells exposed to oxidative stress, so the possibility that the antioxidants were inactivating NF-κB was tested. In fact, we found that the ROS produced by all three different injuries activated NF-κB, while Tat-MT, Tat-SOD, and even more so the antioxidants combined, inhibited NF-κB activation (S4 Fig). Antioxidants also reduced iNOS expression and resultant increases in NO (data not shown). Collectively, these results indicate that antioxidants, if they are given effectively, oppose multiple redundant ROS-mediated signaling mechanisms, which are also associated with the increases in the expression of components of the RAGE and AGII signaling pathways.

## Discussion

To prove whether effective delivery of endogenous antioxidants to renal tissues may ameliorate underlying pathophysiology of DN, we opted to apply CPP technologies in order to study the ameliorative effect of our Tat-fused MT and SOD proteins in OLETF rats. In OLETF rats, treatment with the fusion-antioxidants appeared to cause in a lowered expression of ROS and subsequent transduction signals in renal cells including MCs delaying the clinical progression to DN. Although the overall magnitude of the protection from proteinuria development appeared to be small, a series of immunohistochemical and biochemical tests showed a definitive protective role for this treatment. Moreover, MT and SOD, delivered to MCs also inhibited different kinds of oxidative injuries and dampened the expression of the downstream signals. In accordance with our experiment, overproduction of ROS leading to oxidative stress in renal tissues, especially in MCs, has been believed to be a causative factor in the etiology of DN [5, 8]. Therefore, antioxidant therapy for diabetes has been frequently studied as a potential modality for the prevention of DN, but results are not consistent as it is difficult to maintain a consistent level of circulating antioxidants and adequate tissue distribution and there is a dearth of suitable antioxidants appropriate for therapeutic application [20]. Instead, a many studies explored strategies and options to increase antioxidant activity in renal tissues in order to reduce cytotoxic damage caused by increased levels of ROS, NO, and cytokines present in diabetes [6, 21]. A strategic plan to deliver an endogenous broad spectrum antioxidant more efficiently or to induce it deliberately could be an alternative option. MT and SOD are good candidate endogenous free-radical scavengers, albeit with limited membrane permeability. We found that continuous, prolonged (16 weeks) and periodic (every 3 days) exposure to MT and SOD proteins exploiting a new delivery technique led to a considerable decrease in levels of microalbuminuria in accordance with the decreases in nitrotyrosine positive area. Although improvement of hyperglycemia *per se* as a mechanism to prevent DN by the treatment of MT and SOD due to nonspecificity of Tat-fusion protein strategy could not be ruled out, this was not the apparent case in our real *in vivo* experiment (Table 1) and we assume that there is a direct protective effect of antioxidants against renal pathology. Therefore, a strategy to induce a nonspecific antioxidant for a considerable period of time and to deliver it efficiently may be an effective candidate for preventing DN.

The potential of Tat as a carrier has been studied in various cell types and tissues [16–18]. In spite of the disadvantage of non-specificity, HIV-1-derived Tat mediates the membrane translocation of many peptides both *in vitro* and *in vivo*, and the functions of the Tat-fused peptides are preserved [16, 17]. Applying this protein transduction technology, we have demonstrated that antioxidants were successfully introduced into renal cells (MCs) and are able to protect the cells from various types of injury related to oxidation *in vitro*. Without the aid of Tat delivery, antioxidant peptides cannot gain substantial entry into the MCs. This work also confirms that our strategy for the intracellular delivery of these fusion proteins preserve their functionality as antioxidants. However, despite efficient short-term delivery, our strategy might suffer from the generation of an immune response to the delivered protein thus deccrasing the effectiveness over time. To circumvent this disadvantage, we conducted several studies to attempt to minimize the amount of potentially immunogenic fusion product that would be available. Finally, we believe we have optimized the appropriate concentration and amount of injected protein to minimize the immune response. Our results show that 16 weeks of prolonged combination treatment of Tat-MT (3 mg/kg) and Tat-SOD (3 mg/kg) every 3 days is sufficient to significantly decrease ME, while dampening glomerular inflammation and fibrosis and significantly attenuating microalbuminuria in diabetic OLETF rats.

Apart from the injuries to thetubulointerstitium and vasculature, oxidative stress mainly gives rise to mesangial lesions characterized by ME and GBM thickening in patients with DN [10]. This mesangial matrix accumulation has been shown to be induced by different ROS injuries causing changes in the amounts and constituents of the ECM including the fibrillar proteins and glycoproteins such as types I, III and IV collagen, fibronectin and laminin. Expression and activity of the ECM regulatory enzymes, such as the matrix metalloproteinases and tissue inhibitors of matrix metalloproteinases as well as growth factors, such as PDGF, TGF-β1, and CTGF, are affected as well [4, 8, 11]. Also, MCs are essential cellular mediators of the intercellular, interstial space in the kidney and play a crucial role in mediating ME. Experiments using primary cultures of MCs as surrogates have shown that oxidative stress is a primary factor in the etiology of DN. Other research has shown that the renal functional and structural changes found in patients with DN are also associated with increased expression of TGF-β, CTGF and VEGF and that aggravated inflammation, mostly in MCs, is pathogenic for glomerulosclerosis and proteinuria. However, endogenous antioxidants are known to protect MCs against free radical oxidation and ROS cytotoxicity.

In our study, the renoprotective effect of the MT and SOD Tat-fusion proteins was associated with decrease of renal profibrotic factors (TGF-β, VEGF and CTGF) as well as reduction of inflammatory molecules (ICAM-1, RAGE, collagen, laminin and fibronectin) via multiple redundant ROS-mediated pathways. We also found that these endogenous antioxidants directly reduced degrees of AGII-induced injuries, which are either due to hemodynamic perturbation or metabolic derangement, and resulted in suppression of NOX4 and down regulation of the synthesis of several inflammatory molecules in the cultured MCs. In addition to its well-known metabolic assaults on the kidneys, hyperglycemia causes dysfunctional autoregulation in glomeruli by stimulating the intra-renal renin-angiotensin aldosterone axis, leading to activation of local AGII production [7, 12]. This might increase glomerular capillary pressure thereby enhancing mechanical stretching of the MCs which then activate ROS signals, amplifying various redundant intracellular pathways and aggravating DN synergistically. In our study of MCs, as expected, AGII, a major contributor to renal injury, dose-dependently increased ROS formation and the expression of inflammatory molecules including AT-1, while the exogenously administered Tat-fused antioxidants significantly reduced these effects. We also showed that prolonged treatment with these antioxidants resulted in improved measurements of urinary albumin excretion and pathology in chronically hyperglycemic OLETF rats.

The biological effects of MT and SOD could be ascribed to their function as free-radical scavengers acting as intracellular antioxidants. Delivery of recombinant SOD protein (or SOD mimetics) into injured renal tissues has already been shown to promote renal repair and enhance functional amelioration following initiation of DN [5]. However, if the SOD were simply provided in solution it would diffuse away rapidly in the body fluids. Hence impractical repeated injections would be needed and ultimately excessive doses might evoke undesirable adverse effects. To circumvent this, many groups are trying to introduce SOD to the renal tissues via amenable drug delivery systems or transplantation of cells applying encapsulation. These maneuvers need to be improved with regard to release, dosing, efficacy and safety. In our experiments we exploited recent CPP technology to circumvent these hurdles. To generalise the beneficial *in vivo* effects of these ROS scavenger, we studied the effect of these novel fusion peptide antioxidants in rat MCs in three different *in vitro* models of ROS-mediated injury, hyperglycemia, AGE and 4-HNE. In these three *in vitro* models, the endogenous antioxidants significantly reduced expression of RAGE and AGII. Although the precise defects in MCs resulting from the different types of injury are not well understood, NF-κB activation by oxidative stress is a likely early event [22], and this view was supported by our own experiments. Although the exact contribution of NFκβ may differ in different clinical situations during DN development, ROS seem to play a crucial role in NFκβ activation [23]. Ha et al. demonstrated increased NFκβ activity in MCs with high glucose concentration and ROS generated under this high glucose condition plays an important role in NFκβ activation [24]. NFκβ is a redox sensitive transcription factor and its activation is an initial signaling event stimulating the activation of other inflammatory pathways that leads to various microvascular damages found in patients with diabetes [25]. Deregulation of cellular downstream molecular cascades and controls secondary to prolonged hyperglycemic environments in the cell leads to endothelium, podocyte, MC as well as tubular epithelial cell injury, which may be the decisive factor in the pathogenesis of DN. MAPK and phosphatidyl inositol–3 kinase have also been implicated in the induction of VEGF and their downstream molecular events are also involved with activation of NFκβ. NFκβ induces the expression of a large number of gene products that influence inflammation, cell proliferation, angiogenesis and cellular adhesion [26].

We also found that the increased oxidative stress resulting from the various mechanisms of ROS injury was accompanied by activation of the MAPK, AMP kinase and possibly the WNT signaling pathways. Besides the classical involvement of MAPK [19, 20], various nutrient-sensing pathways are also important in the development of DN [20, 27]. AMP kinase affects various cellular and lipid metabolic pathways by which it may contribute to renoprotection. In OLETF rats, dysfunctional intra-renal lipid metabolism characterized by increased lipogenesis and decreased β-oxidation is frequently found. Although we do not have the data, reduced renal AMP kinase activity in OLETFs may explain changes in renal lipid metabolism and subsequent lipotoxicity-associated renal damage. Activation of AMP kinase by antioxidant may turn down a lipogenic enzyme, acetyl-CoA carboxylase, and activate the mitochondrial β-oxidation machinery such as carnitine palmitoyl transferase-1. Moreover, similar to our own experiments, ROS have also been shown to activate the WNT signals in the kidney of diabetic animals, which would contribute to the exacerbation of healing pathways via recruiting numerous growth factors involved in dysregulated fibrotic formation of scar tissue [28]. The canonical WNT signaling pathway is found to modulate inflammation, angiogenesis, and fibrosis through up-regulation of ICAM-1, VEGF, TNF-α and CTGF [29]. Blockage of the WNT signaling by antioxidants may stop the progression of oxidative stress, inflammation, dysregulated wound repair and fibrosis.

Antioxidant treatment increased the resistance of MCs to ROS, and altered the activation of NF-κB, MAPK, AMP kinase and WNT signaling pathways, as well as the expression of downstream inflammatory regulatory cytokines. These antioxidants almost abolished the increased expression of ICAM-1 and CTGF, as well as of laminin and fibronectin, the final constituents of ECM expansion in cultured MCs. Furthermore, they decreased the activity of MMP-9. All these *in vitro* changes in apoptosis and signaling pathways were supported and confirmed by the results of immunoblotting of renal tissue after 16 weeks of treatment with the combined Tat-fused antioxidants (Fig 3). That combination significantly reduced various ROS-mediated injuries and resultant renal damage *in vivo* and *in vitro* by inhibiting the activation of redundant signals and their downstream inflammatory mediators.

Although we focused on MCs, another important sentinel, podocytes or glomerular visceral epithelial cells are also crucial candidates for study. They are highly specialized and produce foot processes, which interdigitate between cells of the glomeruli, leaving filtration slits [30]. They also synthesize matrix proteins in the GBM, including type IV collagen, laminin, entactin and agrin [4, 26]. Although we have not studied the effect of antioxidants on podocytes in the diabetic model of OLETF rats *per se*, hemodynamic stress leading to overproduction of local growth factors induces glomerular hypertrophy and increase type IV collagen synthesis in podocytes similarly to what is observed in MCs. It can be hypothesized that the activation of similar signal transduction paths and subsequently similar changes in the metabolome of podocytes during DN development may be like those demonstrated in the MCs [3, 4].

In summary, our results reveal that our Tat-fusion constructs with the enzymatic anti-oxidants, MT and/or SOD, either alone but most significantly in combined therapy, inhibited the effects of three different oxidative injuries (hyperglycemia, AGE and 4-HNE exposure), and changes in the pattern of downstream signaling in MCs. Importantly, the enzymatic antioxidant treatment in combination for a prolonged period markedly reduced oxidative injuries and delayed the progression to DN, and presumably, renal failure, in OLETF rats. The renoprotective effects of these novel fusion polypeptide, enzymatically functional, antioxidants in DN may be related to a decrease in concentration of intracellular and extracellular ROS themselves as well as a beneficial alteration of triggers presented to the downstream signal receptors and pathways. We conclude that this effective novel delivery mechanism for combination antioxidant therapy has clearly demonstrated a role for antioxidant therapy, which offers renoprotection and facilitates the repair of damage in patients with DN. Diabetes predisposes individuals to excessive oxidative stress because the antioxidant defense systems are overwhelmed in hyperglycemia, and the resulting unchecked injuries caused by ROS are likely causative factors in developing the formidable complications of diabetes. This work demonstrates that this novel means of antioxidant supplementation can inhibit many of the diabetes-associated alterations that have been attributed to increased concentrations of ROS and NO in hyperglycemia. Since these fusion-protein antioxidants appear to be a novel and promising approach to examining and possibly treating the effects of oxidative stress on the development of diabetic complications such as DN, further study is certainly warranted.

## Supporting Information

S1 Fig Transduction of Tat-fusion proteins into primary cultured mesangial cells and kidney tissue *in vivo*. (A-C) Schematic presentation of Tat-MT, Tat-SOD and Tat-GFP construct subcloned in expression vectors and respective protein blot after expression and purification. (D) Primary cultured mesangial cells (MCs) were incubated for 1 h with1 μmol/l of MT, Tat-MT, SOD, Tat-SOD or Tat-GFP. Protein-matched MC extracts were analyzed by Western blotting using anti-rabbit poly-histidine, anti-goat MT, anti-rabbit SOD and anti-rabbit β-actin antibody. (D) Cellular localization of MT, Tat-MT, SOD, Tat-SOD and Tat-GFP in MCs 1 h after treatment with each protein. Confocal microscopic images of cells stained for poly-histidine (green) and polyclonal MT or SOD antibody (red) are shown (scale bar, 50 μm; original magnification, ×400). (E) Transduction of Tat-fusion proteins into renal tissues was analyzed by Western blotting and immunohistochemistry. Diabetic OLETF rats at 20 week of age were injected *i*.*p*. with a single treatment of 3 mg/kg of Tat-GFP or the same amount of antioxidants in combination. Tissues were dissected from the rats 4 d after transduction and processed for Western blotting and immunohistochemistry using anti-rabbit poly-histidine antibody and anti-mouse β–actin. LETO rats or OLETF rats without transduction were used as histological controls. Results are representative of three separate experiments.
(TIF)

S2 Fig Dose-dependent reduction of ROS and inflammatory molecular induction after AGE injury by transduction of fusion proteins. MCs were incubated with Tat-MT, Tat-SOD or antioxidants in combination at indicated concentrations, and subsequently treated with 400 μg/ml of AGE. Control cells were treated with 400 μg/ml of BSA. β-actin mRNA expression served as a standard. RAGE and fibronectin levels were investigated by Western blotting. Protein-matched cell extracts were probed with polyclonal antibodies and α-tubulin served as loading control. Data are representative of three experiments performed on different days.
(TIF)

S3 Fig Dose-dependent increases in expression of inflammatory molecules by angiotensin II (AGII) and their inhibition by the transduced Tat-MT. (A) Cells were incubated in the absence (control) or presence of 1 ~ 1000 nmol/l AGII for 24h. Expression of inflammatory molecules was measured by RT-PCR (RAGE, AT-1 and collagen IV) or Western blotting (RAGE and fibronectin). (B) Inhibition of inflammatory molecule expression by Tat-MT. Cells were transduced with MT or Tat-MT and subsequently treated with 1000 nmol/l AGII for 24h, and inflammatory molecule expression was determined as in A. β-actin served as loading control. Data are representative of three experiments performed on different days. Abbreviation; AGII: Angiotensin II.
(TIF)

S4 Fig Influence of fusion proteins on activation of MAP kinase, AMP kinase, Wnt and NF-κB after various damages. A) MCs extracts were prepared and analyzed for MAP kinase and AMP kinase activation by immunoblotting using phospho-specific antibodies against MAPK proteins (p38, ERK and JNK) and AMPK proteins. α-tubulin served as loading control. (B) Wnt signal expression in MCs was investigated by Western blotting (total and phospho-LRP6, β-catenin, total and phospho-GSK3β and VEGF). (C) Protein-matched nuclear and cytosolic extracts of the MCs were analyzed by Western blotting using anti-mouse p65 antibody and anti-rabbit α-tubulin antibody. (D) Nuclear extracts were mixed with a double-stranded ^32^P-labeled oligonucleotide encoding the decameric consensus sequence of NF-κB and separated by PAGE. Data are representative of three experiments performed on different days. Abbreviations; LG: low glucose (5.5 mmol/l), NG: normal glucose (11.1 mmol/l), HG: high glucose (30 mmol/l), TGFP: Tat-GFP, TMT: Tat-MT, TSOD: Tat-SOD, TMTS: Tat-MT-Tat-SOD combination. Data are expressed as mean ± SEM (n = 3). ^a^ *p*<0.05, ^b^ *p*<0.01, ^c^ *p*<0.001 between the diverse injury groups.
(TIF)

S1 File The ARRIVE Guidelines Checklist Animal Research: Reporting In Vivo Experiments. (DOCX)

S1 Table Transcript and sequence of each primer used in RT-PCR. (DOCX)
