# Supplementary figures and images for: Effective Delivery of Endogenous Antioxidants Ameliorates Diabetic Nephropathy
Source: PLoS One. 2015 Jun 26;10(6):e0130815. doi: 10.1371/journal.pone.0130815 (PMC4483240; doi:10.1371/journal.pone.0130815)

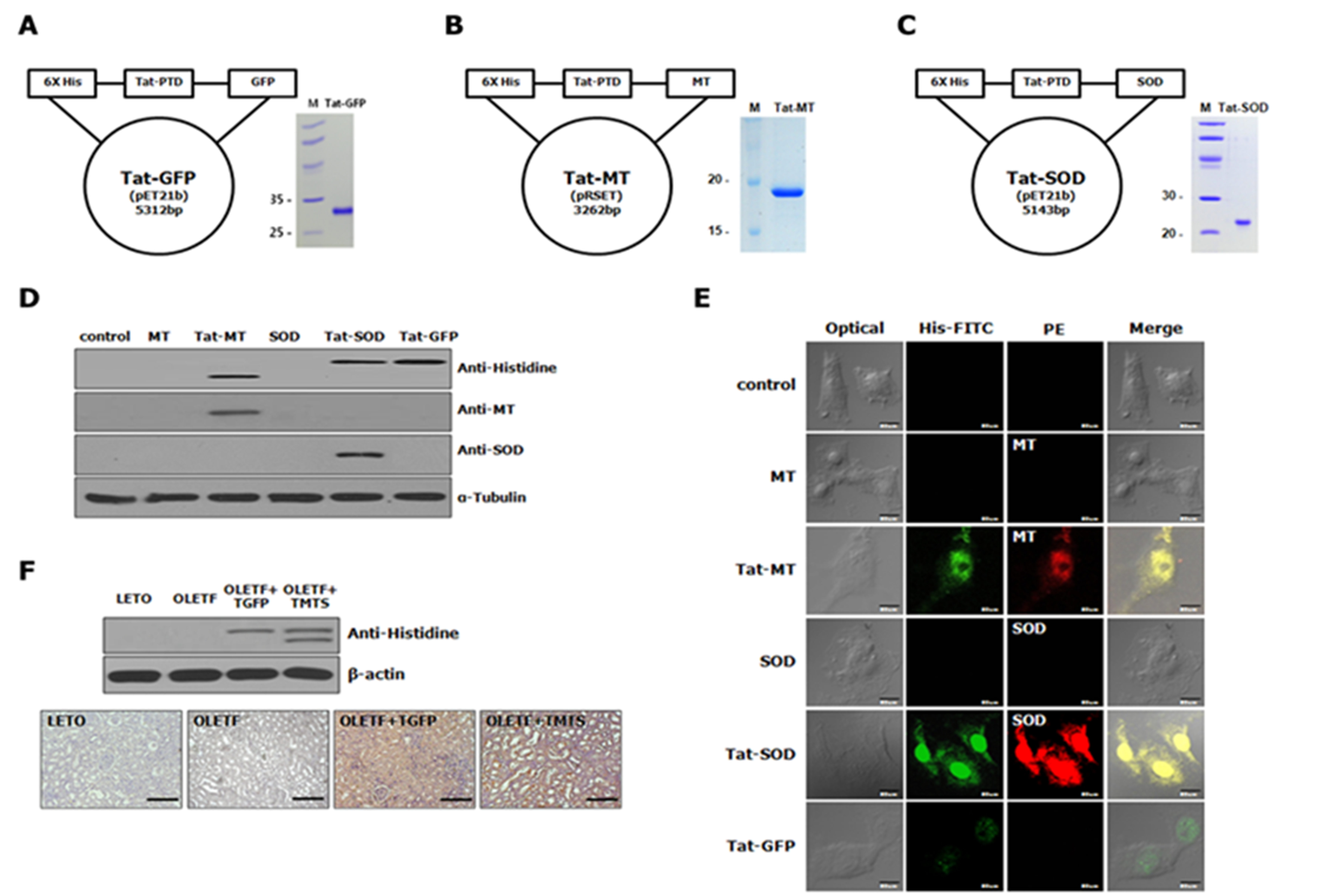

Supplement: S1 Fig — (A-C) Schematic presentation of Tat-MT, Tat-SOD and Tat-GFP construct subcloned in expression vectors and respective protein blot after expression and purification. (D) Primary cultured mesangial cells (MCs) were incubated for 1 h with1 μmol/l of MT, Tat-MT, SOD, Tat-SOD or Tat-GFP. Protein-matched MC extracts were analyzed by Western blotting using anti-rabbit poly-histidine, anti-goat MT, anti-rabbit SOD and anti-rabbit β-actin antibody. (D) Cellular localization of MT, Tat-MT, SOD, Tat-SOD and Tat-GFP in MCs 1 h after treatment with each protein. Confocal microscopic images of cells stained for poly-histidine (green) and polyclonal MT or SOD antibody (red) are shown (scale bar, 50 μm; original magnification, ×400). (E) Transduction of Tat-fusion proteins into renal tissues was analyzed by Western blotting and immunohistochemistry. Diabetic OLETF rats at 20 week of age were injected i.p. with a single treatment of 3 mg/kg of Tat-GFP or the same amount of antioxidants in combination. Tissues were dissected from the rats 4 d after transduction and processed for Western blotting and immunohistochemistry using anti-rabbit poly-histidine antibody and anti-mouse β–actin. LETO rats or OLETF rats without transduction were used as histological controls. Results are representative of three separate experiments. (TIF) [file pone.0130815.s001.tif]

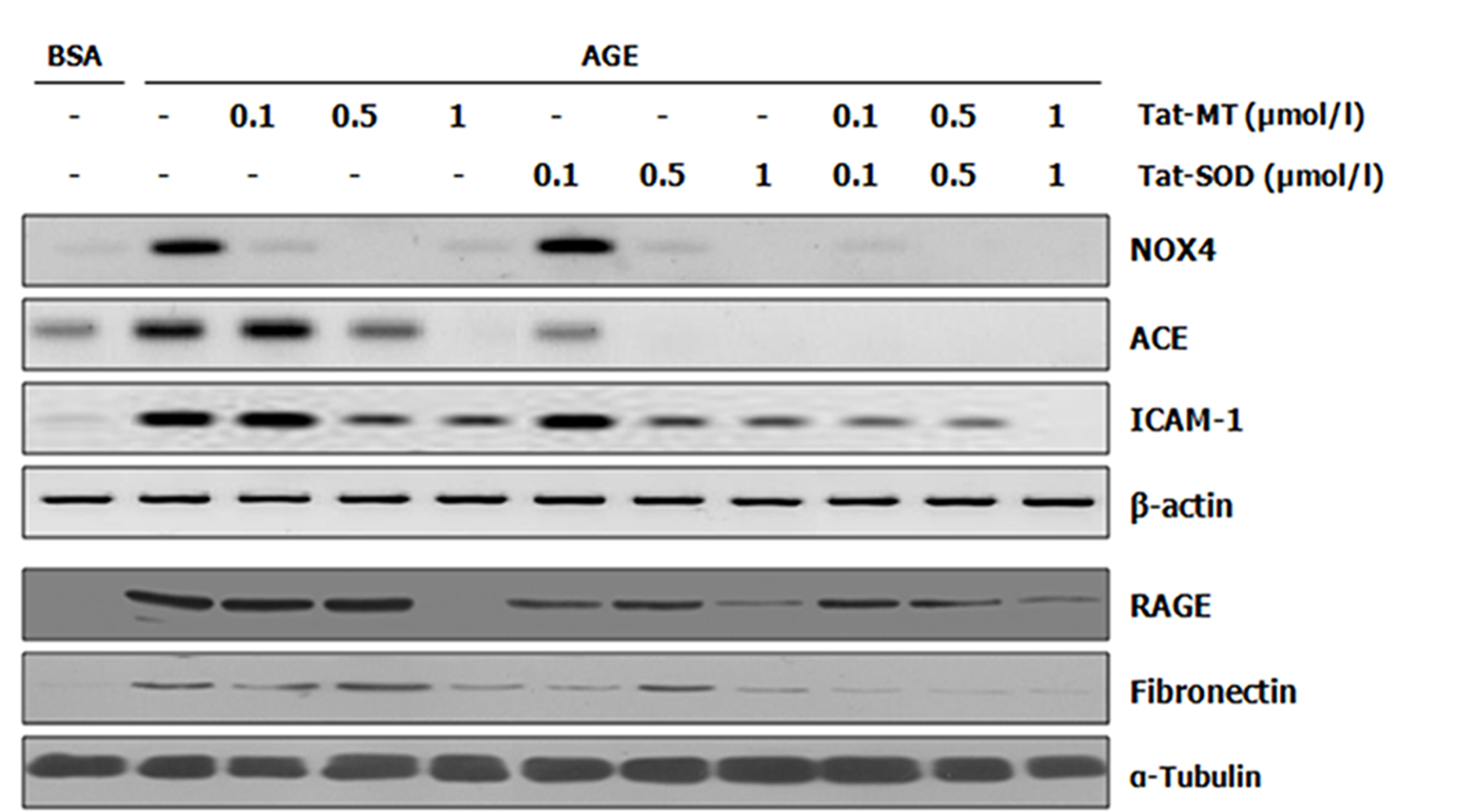

Supplement: S2 Fig — MCs were incubated with Tat-MT, Tat-SOD or antioxidants in combination at indicated concentrations, and subsequently treated with 400 μg/ml of AGE. Control cells were treated with 400 μg/ml of BSA. β-actin mRNA expression served as a standard. RAGE and fibronectin levels were investigated by Western blotting. Protein-matched cell extracts were probed with polyclonal antibodies and α-tubulin served as loading control. Data are representative of three experiments performed on different days. (TIF) [file pone.0130815.s002.tif]

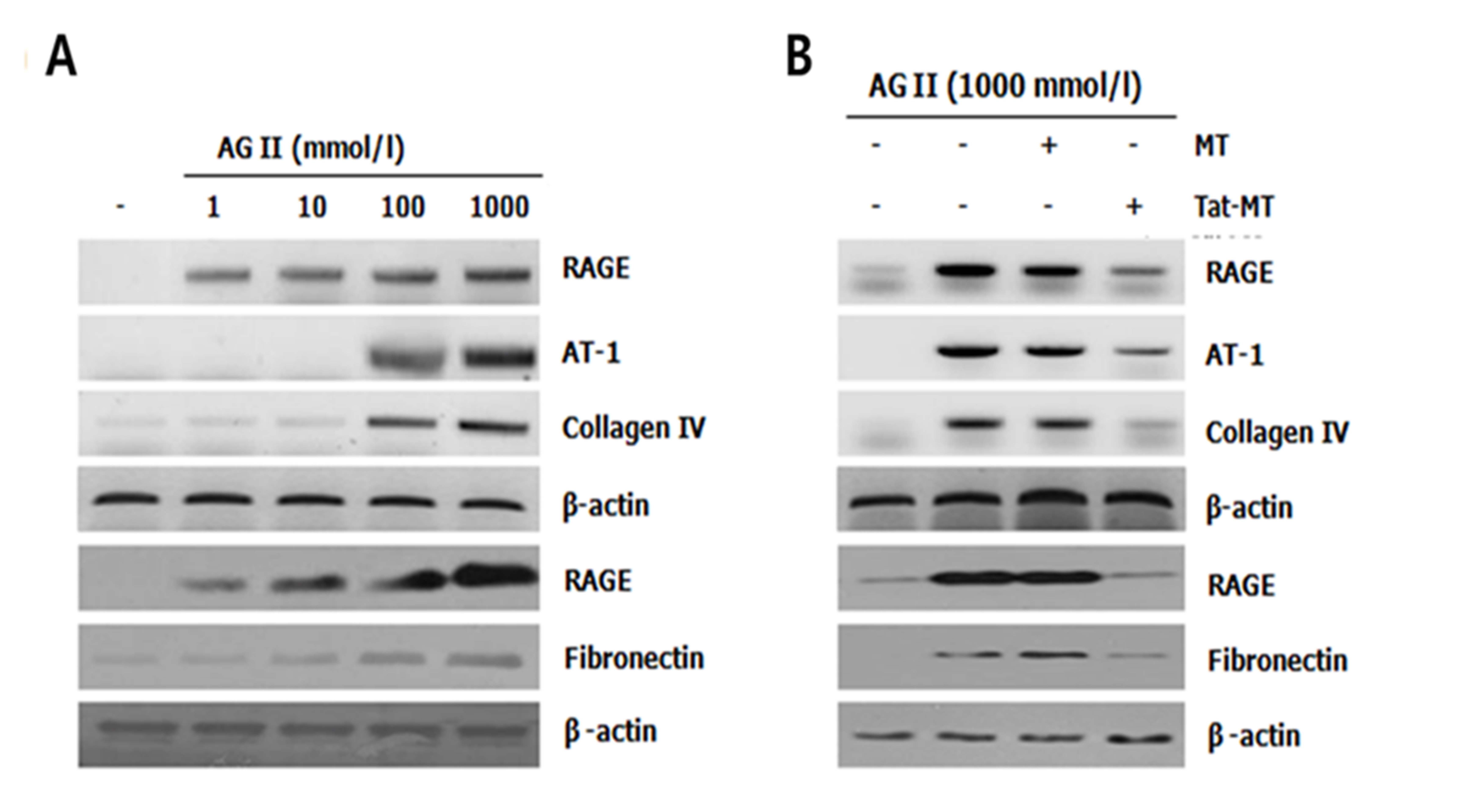

Supplement: S3 Fig — (A) Cells were incubated in the absence (control) or presence of 1 ~ 1000 nmol/l AGII for 24h. Expression of inflammatory molecules was measured by RT-PCR (RAGE, AT-1 and collagen IV) or Western blotting (RAGE and fibronectin). (B) Inhibition of inflammatory molecule expression by Tat-MT. Cells were transduced with MT or Tat-MT and subsequently treated with 1000 nmol/l AGII for 24h, and inflammatory molecule expression was determined as in A. β-actin served as loading control. Data are representative of three experiments performed on different days. Abbreviation; AGII: Angiotensin II. (TIF) [file pone.0130815.s003.tif]

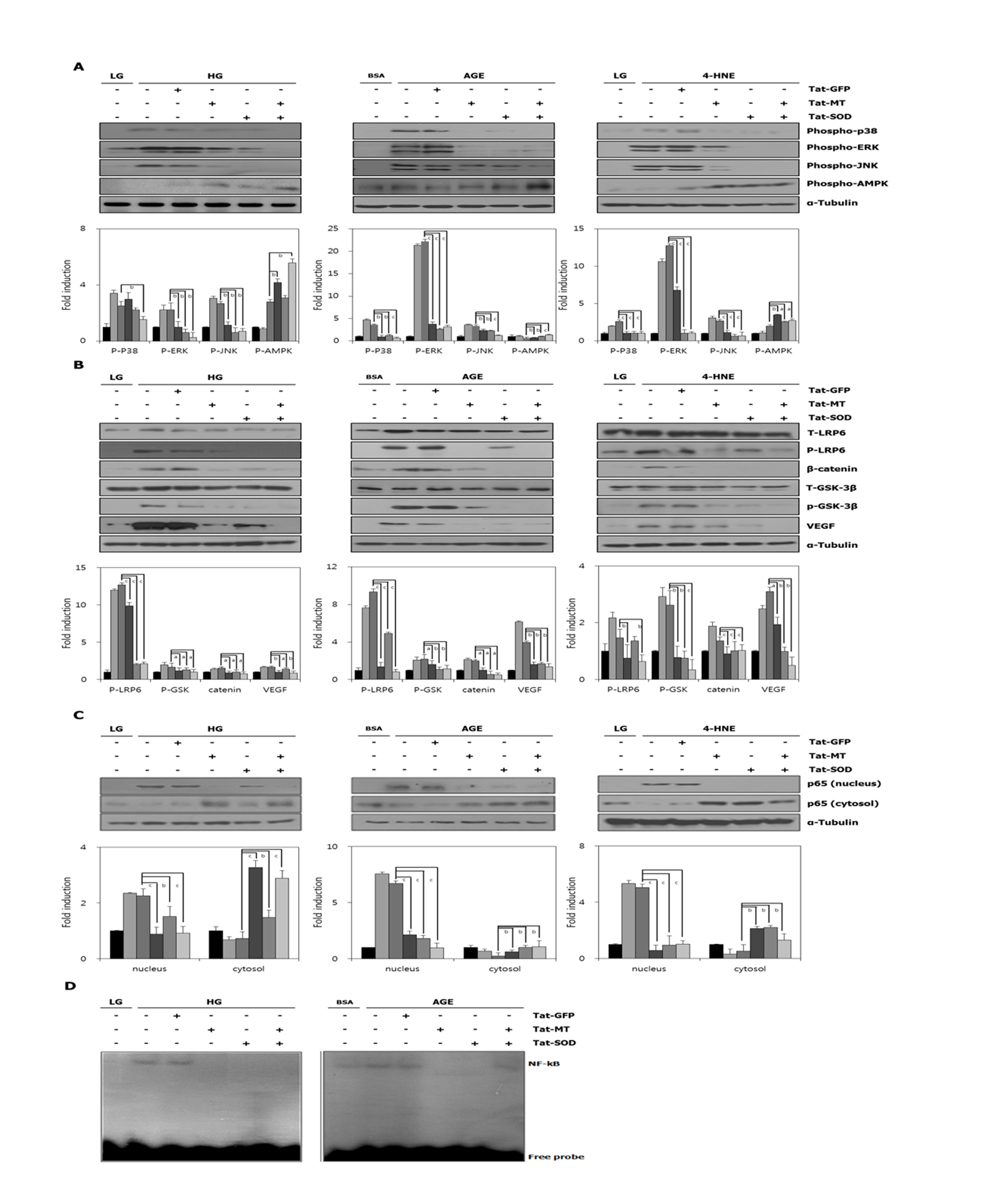

Supplement: S4 Fig — A) MCs extracts were prepared and analyzed for MAP kinase and AMP kinase activation by immunoblotting using phospho-specific antibodies against MAPK proteins (p38, ERK and JNK) and AMPK proteins. α-tubulin served as loading control. (B) Wnt signal expression in MCs was investigated by Western blotting (total and phospho-LRP6, β-catenin, total and phospho-GSK3β and VEGF). (C) Protein-matched nuclear and cytosolic extracts of the MCs were analyzed by Western blotting using anti-mouse p65 antibody and anti-rabbit α-tubulin antibody. (D) Nuclear extracts were mixed with a double-stranded 32P-labeled oligonucleotide encoding the decameric consensus sequence of NF-κB and separated by PAGE. Data are representative of three experiments performed on different days. Abbreviations; LG: low glucose (5.5 mmol/l), NG: normal glucose (11.1 mmol/l), HG: high glucose (30 mmol/l), TGFP: Tat-GFP, TMT: Tat-MT, TSOD: Tat-SOD, TMTS: Tat-MT-Tat-SOD combination. Data are expressed as mean ± SEM (n = 3). a p<0.05, b p<0.01, c p<0.001 between the diverse injury groups. (TIF) [file pone.0130815.s004.tif]
