# Supplementary material for: Effective Delivery of Endogenous Antioxidants Ameliorates Diabetic Nephropathy
Source: PLoS One. 2015 Jun 26;10(6):e0130815. doi: 10.1371/journal.pone.0130815 (PMC4483240; doi:10.1371/journal.pone.0130815)
Supplement: S1 File — (DOCX) [file pone.0130815.s005.docx]

**S1 File. The ARRIVE Guidelines Checklist**

**Animal Research: Reporting In Vivo Experiments**

|  | **ITEM** | **RECOMMENDATION** |
| --- | --- | --- |
| **Title** | **1** | **Provide as accurate and concise a description of the content of the article as possible.** |
|  |  | We did as depicted in the ‘Title’. |
| **Abstract** | **2** | **Provide an accurate summary of the background, research objectives, including details of the species or strain of animal used, key methods, principal findings and conclusions of the study.** |
|  |  | We did as depicted in the ‘Abstact’. |
| **INTRODUCTION** | | |
| **Background** | **3** | **a. Include sufficient scientific background (including relevant references to previous work) to understand the motivation and context for the study, and explain the experimental approach and rationale.**  **b. Explain how and why the animal species and model being used can address the scientific objectives and, where appropriate, the study’s relevance to human biology.** |
|  |  | a. We did as depicted in the ‘Introduction’.  B. The rat model, OLETF (type 2 diabetes model) and LETO (normal control) rats are selected because they are already found to be a ell-established model for the investigation of diabetes and diabetic complication. And the pathophysiology of them resembles human condition. We have already published the studies of diabetes and diabetic complication development using these models that we did not reiterate these in this particular manuscript. |
| **Objectives** | **4** | **Clearly describe the primary and any secondary objectives of the study, or specific hypotheses being tested.** |
|  |  | Primary objective: We investigated whether effective delivery of potent endogenous antioxidants, MT and SOD, which have limited membrane permeability enables the clinical improvement of diabetic nephropathy.  Secondary objective: We also studied the effect of antioxidant on primary rat mesangial cells (MCs), which are important constituents of renal glomeruli, from various forms of oxidative damage. |
| **METHODS** | | |
| **Ethical statement** | **5** | **Indicate the nature of the ethical review permissions, relevant licences (e.g. Animal [Scientific Procedures] Act 1986), and national or institutional guidelines for the care and use of animals, that cover the research.** |
|  |  | This study was carrired out in strict accordance with the recommendations in the Guide for the Care and Use of Laboratory Animals of the National Institutes of Health (NIH, publication No. 85-23 m, revised 1996). The protocol was approved by the Institutional Animal Care and Use Committee (IACUC) at the University of Hanyang (Permission Number: HY-IACUC-09-021). |
| **Study design** | **6** | **For each experiment, give brief details of the study design including:**  **a. The number of experimental and control groups.**  **b. Any steps taken to minimise the effects of subjective bias when allocating animals to treatment (e.g. randomisation procedure) and when assessing results (e.g. if done, describe who was blinded and when).**  **c. The experimental unit (e.g. a single animal, group or cage of animals).**  **A time-line diagram or flow chart can be useful to illustrate how complex study designs were carried out.** |
|  |  | a. Controls: LETO control rats (n=10),  Experimental groups: OLETF rats without transduction group (n=10), a Tat-GFP group (n=9) and a Tat-MT-Tat-SOD combination group (n=8)  b. As depicted in the ‘Method’, all the diabetic OLETF rats were randomly divided into three groups: OLETF rats without transduction group (n=10), a Tat-GFP group (n=9) and a Tat-MT-Tat-SOD combination group (n=8). Owing to this randomization, the clinical characteristics are not different between the groups before the protein treatment (Table 1).  c. All rats housed one per cage were kept at controlled temperature (23 ± 2°C) and humidity (55 ± 5%) with a 12 h light/dark cycle, and allowed free access to standard rat chow. The description of the experiments was given in a sequential fashion. |
| **Experimental**  **procedures** | **7** | **For each experiment and each experimental group, including controls, provide precise details of all procedures carried out. For example:**  **a. How (e.g. drug formulation and dose, site and route of administration, anaesthesia and analgesia used [including monitoring], surgical procedure, method of euthanasia). Provide details of any specialist equipment used, including supplier(s).**  **b. When (e.g. time of day).**  **c. Where (e.g. home cage, laboratory, water maze).**  **d. Why (e.g. rationale for choice of specific anaesthetic, route of**  **administration, drug dose used).** |
|  |  | a. OLETF rats were injected every 3 days with 3 mg/kg of Tat-fusion protein (Tat-GFP group (Tat-GFP); Tat-MT-Tat-SOD combination group (Tat-MT 3 mg/kg and Tat-SOD 3 mg/kg) for 16 weeks  b. When: in the morning (from 9:00 – 11:00 am)  c. Where: Experimental laboratory at the Hanyang university  d. Why: At the end of the experiment, all animals were euthanized with ketamine, and kidney tissues were removed. The animals are anesthetized by an intraperitoneal injection of ketamine (75mg/kg), which will provide 30~60 minutes of anesthesia. |
| **Experimental**  **animals** | **8** | **a. Provide details of the animals used, including species, strain, sex, developmental stage (e.g. mean or median age plus age range) and weight (e.g. mean or median weight plus weight range).**  **b. Provide further relevant information such as the source of animals, international strain nomenclature, genetic modification status (e.g. knock-out or transgenic), genotype, health/immune status, drug or test naïve, previous procedures, etc.** |
|  |  | a. Male LETO and OLETF rats (20 to 36 weeks), weighting 500~ 600g were used in this study.  b. Otsuka Long Evans Tokushima Fatty (OLETF) rat is a useful animal model of type II diabetes with obesity. It shows a clinically relevant phenotypes of diabetes such as, hyperinsulinemia, hyperglycemia, insulin resistance, hypertriglycemia, mild obesity. |
| **Housing and husbandry** | **9** | **Provide details of:**  **a. Housing (type of facility e.g. specific pathogen free [SPF]; type of cage or housing; bedding material; number of cage companions; tank shape and material etc. for fish).**  **b. Husbandry conditions (e.g. breeding programme, light/dark cycle, temperature, quality of water etc for fish, type of food, access to food and water, environmental enrichment).**  **c. Welfare-related assessments and interventions that were carried out prior to, during, or after the experiment.** |
|  |  | a. One animal per cage  b. Standard atmospheric pressure, 12 h light/dark cycle,  controlled temperature (23 ± 2°C) and humidity (55 ± 5%), spontaneus water intake, regular feeding, cleaning and disinfection.  c. The animals were anesthetized by intraperitoneal injection of ketamin (75mg/kg), which provided 30~60 minutes of anesthesia. During anesthesia, kidney was removed rapidly for morphological and molecular examinations and all efforts were made to minimize suffering. |
| **Sample size** | **10** | **a. Specify the total number of animals used in each experiment, and the number of animals in each experimental group.**  **b. Explain how the number of animals was arrived at. Provide details of any sample size calculation used.**  **c. Indicate the number of independent replications of each experiment, if relevant.** |
|  |  | a. LETO controls (n = 10), OLETF (n=10), Tat-GFP (n=9), Tat-MT-Tat-SOD (n=8)  b. The numbers proposed were sufficient for the comparison between the groups based on our previous experiences and analysis.  c. Not relevant |
| **Allocating animals to experimental groups** | **11** | **a. Give full details of how animals were allocated to experimental groups, including randomisation or matching if done.**  **b. Describe the order in which the animals in the different experimental groups were treated and assessed.** |
|  |  | a. N/A  b. N/A |
| **Experimental outcomes** | **12** | **Clearly define the primary and secondary experimental outcomes assessed (e.g. cell death, molecular markers, behavioural changes).** |
|  |  | Primary outcomes: 24 hour urine microalbumin quantification (To study whether effective delivery of MT and SOD enables the clinical improvement of diabetic nephropathy).  Secondary outcomes: RAGE, Angiotensin II, NADPH Oxidase (NOX4), Collagen IV, Fibronectin, TGFB, CTGF, et al. (To study the effect of antioxidant on primary rat mesangial cells from various forms of oxidative damage). |
| **Statistical methods** | **13** | **a. Provide details of the statistical methods used for each analysis.**  **b. Specify the unit of analysis for each dataset (e.g. single animal, group of animals, single neuron).**  **c. Describe any methods used to assess whether the data met the assumptions of the statistical approach.** |
|  |  | a. Data were analyzed by one-way or repeated measures of analysis of variance (ANOVA).  b. Group of animals  C. Data are presented as means ± SEM. Differences in mean values were tested by Student’s t-test using SPSS for Windows (version 18.0; SPSS, Chicago, IL). Data were analyzed by one-way or repeated measures of analysis of variance (ANOVA). P values < 0.05 were considered statistically significant. |
| **RESULT** | | |
| **Baseline data** | **14** | **For each experimental group, report relevant characteristics and health status of animals (e.g. weight, microbiological status, and drug or test naïve) prior to treatment or testing. (This information can often be tabulated).** |
|  |  | We did as depicted in Table 1. |
| **Numbers analysed** | **15** | **a. Report the number of animals in each group included in each analysis. Report absolute numbers (e.g. 10/20, not 50%2).**  **b. If any animals or data were not included in the analysis, explain why.** |
|  |  | a. Comparison between 3 different groups of OLETF and LETO: OLETF rats without transduction group (10/10), a Tat-GFP group (9/9) and a Tat-MT-Tat-SOD combination group (8/8)  b. All animals were included in the statistical analysis |
| **Outcomes and estimation** | **16** | **Report the results for each analysis carried out, with a measure of precision (e.g. standard error or confidence interval).** |
|  |  | Yes, we did. All data are presented as means ± standard errors. |
| **Adverse events** | **17** | **a. Give details of all important adverse events in each experimental group.**  **b. Describe any modifications to the experimental protocols made to reduce adverse events.** |
|  |  | No, we have not found any. |
| **DISCUSSION** | | |
| **Interpretation/ scientific implications** | **18** | **a. Interpret the results, taking into account the study objectives and hypotheses, current theory and other relevant studies in the literature.**  **b. Comment on the study limitations including any potential sources of bias, any limitations of the animal model, and the imprecision associated with the results.**  **c. Describe any implications of your experimental methods or findings for the replacement, refinement or reduction (the 3Rs) of the use of animals in research.** |
|  |  | a. Yes, we did. It was described in the ‘Discussion’.  b. Since the Tat-fusion protein treatment was systemic, the beneficial influence of the protein treatment could be affected by the ameliorative effect of the protein to other organs such as pancreas. It was discussed in the ‘Discussion’.  c. Although it was inevitable to use of animals in this study as a surrogate to human study, now we all agree that antioxidant treatment may ameliorate the development of diabetic nephropathy, if it is given in an effective way. No other further animal experiments will be needed to study the beneficial effect of any antioxidant to reduce the development of diabetic nephropathy. |
| **Generalisability/ translation** | **19** | **Comment on whether, and how, the findings of this study are likely to translate to other species or systems, including any relevance to human biology.** |
|  |  | If we find an effective carrier to transduce the antioxidant intracellularly, appropriate antioxidant treatment in man may prevent of delay the development diabetic complications such as diabetic nephropathy. |
| **Funding** | **20** | **List all funding sources (including grant number) and the role of the funder(s) in the study.** |
|  |  | This research was supported by the Basic Science Research Program through the National Research Foundation of Korea (NRF) funded by the Ministry of Education, Science and Technology (2010-0010898) and the Korea Health 21 R&D Project, Ministry of Health and Welfare, Republic of Korea (A102065).  Future study will be needed to find appropriate cell penetrating peptides for humans to facilitate translation into humans. |
