# Supplementary material for: Effective Delivery of Endogenous Antioxidants Ameliorates Diabetic Nephropathy
Source: PLoS One. 2015 Jun 26;10(6):e0130815. doi: 10.1371/journal.pone.0130815 (PMC4483240; doi:10.1371/journal.pone.0130815)
Supplement: S1 Table — (DOCX) [file pone.0130815.s006.docx]

S1 Table. Transcript and sequence of each primer used in RT-PCR.

|  | **Sense (5’🡪3’)** | **antisense (5’🡪3’)** |
| --- | --- | --- |
| RAGE | CAT CAG GGT CAC AGA AAC CG | GGA AGC TGA AGG AGA CAG GG |
| ACE | GCC ACA TCC AGT ATT TCA TGC AGT | AAC TGG AAC TGG ATG ATG AAG CTG A |
| AT-1 | GGA AAC AGC TTG GTG GTG | TTC TTC CGA ATA CTT TAA GTT |
| NOX 4 | CGG GCC TGA CAG GTG TCT GCA | CTC AGC ACA GTA TAG GCA CA |
| Collagen IV | GTG CGG TTT GTG AAG CAC CG | GTT CTT CTC ATG CAC ACT T |
| ICAM-1 | AGG TAT CCA TCC ATC CCA CA | GCC ACA GTT CTC AAA GCA CA |
| Β-actin | AAG ATG ACC CAG ATC ATG TTT GAG | AGG AGG AGC AAT GAT CTT GAT CTT |
